# CLARK: fast and accurate classification of metagenomic and genomic sequences using discriminative *k*-mers

**DOI:** 10.1186/s12864-015-1419-2

**Published:** 2015-03-25

**Authors:** Rachid Ounit, Steve Wanamaker, Timothy J Close, Stefano Lonardi

**Affiliations:** Department of Computer Science & Engineering, University of California, 900 University Avenue, CA, 92521 Riverside USA; Department of Plant & Botanic Sciences, University of California, 900 University Avenue, CA, 92521 Riverside USA

**Keywords:** Metagenomics, Genomics, Arm/chromosome assignments, Discriminative *k*-mers, Sequence-specific *k*-mers, Chromosome arm, Centromere

## Abstract

**Background:**

The problem of supervised DNA sequence classification arises in several fields of computational molecular biology. Although this problem has been extensively studied, it is still computationally challenging due to size of the datasets that modern sequencing technologies can produce.

**Results:**

We introduce Clark a novel approach to classify metagenomic reads at the species or genus level with high accuracy and high speed. Extensive experimental results on various metagenomic samples show that the classification accuracy of Clark is better or comparable to the best state-of-the-art tools and it is significantly faster than any of its competitors. In its fastest single-threaded mode Clark classifies, with high accuracy, about 32 million metagenomic short reads per minute. Clark can also classify BAC clones or transcripts to chromosome arms and centromeric regions.

**Conclusions:**

Clark is a versatile, fast and accurate sequence classification method, especially useful for metagenomics and genomics applications. It is freely available at http://clark.cs.ucr.edu/.

**Electronic supplementary material:**

The online version of this article (doi:10.1186/s12864-015-1419-2) contains supplementary material, which is available to authorized users.

## Background

The classification problem of determining the origin of a given DNA sequence (e.g., a read or a transcript) in a given set of target sequences (e.g., a set of known genomes) is common to several fields of computational molecular biology. Here, we focus our attention on two applications related to metagenomics and genomics.

In metagenomics, the objective is to study the composition of microbial community in an environmental sample. For example, sequencing of seawater samples has enabled discoveries in microbial diversity in the marine environment [1]. Similarly, the study of samples from the human body has elucidated the symbiotic relationships between the human microbiome and human health [2,3]. Once a metagenomic sample is sequenced, the first task is to determine the identities of the microbial species present in the sample. Several tools are available to classify metagenomic reads against known bacterial genomes via alignment (e.g., [4-7]) or sequence composition (e.g., [8-11]). A recent comparative evaluation of these tools [12] demonstrated that NBC [8] exhibits the highest accuracy and sensitivity at the genus level among [4-6,9]. This study also showed that NBC and other probabilistic methods (e.g., PHYMMBL [5]) as well BLAST-based methods (e.g., MEGAN [4], METAPHYLER [6]) are computationally expensive. Recently, new faster methods have been introduced (e.g., KRAKEN [11]) but their performance still does not meet NBC’s sensitivity. To the best of our knowledge, there is no tool yet that has both a sensitivity comparable to NBC and a speed comparable to KRAKEN. A related group of metagenomic tools, such as METAPHLAN [7] and WGSQUIKR [13] addresses the abundance estimation problem, that is, they estimate from the reads the proportion of each organism present in the sample.

The second application is associated with *de novo* clone-by-clone sequencing and assembly. Given a BAC clone (or a transcript), an objective of a classification problem sometimes is to determine which chromosome (or arm) is the most likely origin of that clone/transcript. The problem assumes that reads for each BAC/transcript as well as reads for each chromosome arm are available, but that the fully-assembled reference genome is not. This is the situation in barley, which we have used for this work, and for many other organisms. In the past, the BAC/transcript assignment problem had been addressed using general-purpose alignment tools (e.g., BLAST [14] or BLAT [15]), as in [16].

In both of these applications the computational problem is the same: given a set of DNA sequences to be classified (henceforth called “objects”) and a set of reference sequences (e.g., genus-level sequences, chromosome arms, etc., henceforth called “targets”), identify which target is the most likely origin of each object based on sequence similarity. Although this problem has been extensively studied, it is still computationally challenging due to the rapid advances in sequencing technologies: cheaper, faster, sequencing instruments can now generate billion of reads in a few days. As the number of objects grows, so does the number of targets, as demonstrated by the exponential growth of GenBank [17]. Given these demands, it is critical for software tools to minimize computational resources (time, memory, I/O, etc) required for analysis.

Here we present CLARK (CLAssifier based on Reduced K-mers), a new tool that can accurately and efficiently classify objects to targets, based on reduced sets of *k*-mers (i.e., DNA words of length *k*). CLARK is the first method able to perform classification of short metagenomics reads at the genus/species level with a sensitivity comparable to that of NBC, while achieving a comparable speed to KRAKEN. In some situations, CLARK can be faster and more precise than KRAKEN at the genus/species level. Unlike tools like LMAT [10], METAPHYLAN, PHYLOPYTHIAS [9], METAPHYLER [6], or NBC, CLARK produces assignments with confidence scores, which are critical to post-process assignments in downstream analyses. Additionally, CLARK is designed to be user-friendly, self-contained (i.e., does not depend on any other tool or library), and multi-core-friendly. CLARK does not need as much disk space as KRAKEN or PHYMMBL. Finally, a “RAM-light” version of CLARK can be run on a memory-limited architecture (such as a 4 GB RAM laptop).

## Results and discussion

We briefly review CLARK’s algorithm before reporting experimental results.

### Target-specific *k*-mers and Classification

During preprocessing, CLARK builds a large index containing the *k*-spectrums of all targets sequences. We recall that a *k*-mer is a DNA word of fixed length *k*, and that the *k*-spectrum of a string *x* is the vector of dimension 4^*k*^ that collects the number of occurences of all possible *k*-mers in *x*. The *k*-spectrum is a succinct (lossy) representation of *x*, which allows sequence comparison (see e.g., [18]). Once all *k*-spectrums of target sequences have been collected in the index, CLARK removes any common *k*-mers between targets (see Methods section).

Henceforth, we call the remaining *k*-mers either *target-specific* or *discriminative*, because they represent genomic regions that uniquely characterize each target. Finally, an object is assigned to the target with which it shares the highest number of *k*-mers.

CLARK offers two modes of execution. The first mode (henceforth named “full”) outputs for each object the number of hits against all the targets and the confidence score of the assignment (which is a number 0.5–1.0). The second mode (“default”) employs sampling to reduce the number the target-specific *k*-mers for classification, and outputs assignments without any detailed statistics so that the output size is significantly reduced (see Methods section for more details). The default mode is slightly less accurate, but it is faster.

### Metagenomics classification

Inputs to this classification task are (1) NCBI/RefSeq databases of known bacterial genomes (targets) and, either (2A) the set of metagenomic reads used in [11] and the set of simulated long reads from “simHC” [19], or (2B) the set of real metagenomic reads from the Human Microbiome Project (objects). The Human Microbiome Project data are freely accessible [2,3].

At the time we carried out the experiments the NCBI/RefSeq database was composed of 2,752 complete bacterial genomes, distributed into 695 distinct genera, or 1,473 species. The total length of all these bacterial genomes was about 9.5 Gbp. The average size of a genome was about 3.5 Mbp.

In the first experiment, we used three microbial metagenomics datasets called “HiSeq”, “MiSeq” and “simBA-5” that were introduced in [11]. According to [11], “the HiSeq and MiSeq metagenomes were built using twenty sets of bacterial whole-genome shotgun reads. These reads were found either as part of the GAGE-B project [20] or in the NCBI Sequence Read Archive. Each metagenome contains sequences from ten genomes (see Additional file 1: Table S1 in [11] for the list of genomes). For these metagenomes, 10% of their sequences were selected from each of the ten component genome data sets (i.e., each genome had equal sequence abundance)”. The set “simBA-5” included “simulated bacterial and archaeal reads, and was created with an error rate five times higher than” the default (see [11]). We also analyzed the set “simHC” of synthetic reads [19], which simulates high complexity communities lacking dominant populations. SimHC contains 113 sets of reads from various microbial genomes. From simHC, we selected arbitrarily twenty distinct genomes, and extracted the first 500 reads for each genome to build a total of 10,000 reads (see Additional file 1: Table S4). We called this latter dataset “simHC.20.500”.

For the experiments below we used the “HiSeq”, “MiSeq” (which can be considered set of read of low/medium complexity), “simBA-5” from [11] and “simHC.20.500” (which can be considered set of reads of high complexity). Each of these sets contains 10,000 reads. The average read length in HiSeq was 92 bp, 156 bp in MiSeq, and 951 bp in simHC.20.500. In simBA-5, all reads are 100 bp long.

In the second experiment, we have arbitrarily chosen three metagenomic samples selected from the Human Microbiome Project [2,3]. The three samples we used were SRS015072 (mid-vagina) containing 572 thousand paired-end reads, SRS019120 (saliva) containing 4.3 million paired-end reads, and SRS023847 (nose) containing 5.2 million paired-end reads.

#### HiSeq, MiSeq, simBA-5 and simHC.20.500

We used CLARK to classify the reads in the four datasets described above and compared its classification results against the state-of-the-art methods, namely NBC [8], which we chose for its high accuracy (currently the most sensitive metagenomics classifier, according to [12]), and KRAKEN, which we chose due to its high speed (currently the fastest metagenomics classifier, according to [11]) and its high precision at the genus level.

We classified the reads (i) against 695 genus-level targets (Table 1) and (ii) against 1473 species-level targets (Table 2).

**Table 1:** **Genus-level classification accuracy and speed of** CLARK, KRAKEN **, and** NBC **for four simulated metagenomes and several** ***k*** **-mer length**

	***k***	**HiSeq**			**MiSeq**			**simBA-5**			**simHC.20.500**		
		***Prec***	***Sens***	***Speed***	***Prec***	***Sens***	***Speed***	***Prec***	***Sens***	***Speed***	***Prec***	***Sens***	***Speed***
NBC	15 ^∗^	**82.57**	**82.57**	0.008	**81.00**	**81.00**	0.007	**97.69**	**97.69**	0.007	**99.40**	**99.40**	0.005
	13 ^∗^	78.85	78.85	0.011	77.70	77.70	0.009	92.41	92.41	0.010	98.57	98.57	0.006
	11 ^∗^	58.97	58.97	**0.020**	64.43	64.43	**0.016**	46.10	46.10	**0.017**	86.83	86.83	**0.008**
Clark(full)	31	**99.26**	77.78	**541**	**95.33**	77.69	**435**	98.88	89.67	**591**	**99.68**	**99.42**	121
	27	98.98	79.88	538	93.50	78.57	433	**98.90**	93.09	585	99.67	**99.42**	**122**
	23	97.33	81.97	530	90.06	80.02	426	98.71	94.54	559	99.59	**99.42**	119
	20	87.00	**82.87**	532	82.45	**80.19**	420	97.38	**94.80**	549	99.43	99.41	115
Kraken	31	**99.26**	77.76	**2,332**	**95.50**	77.59	**1,361**	98.28	89.35	**1,976**	96.83	96.55	**237**
	27	99.01	79.85	2,048	93.91	78.47	1,240	**98.31**	92.73	1,917	**96.85**	96.57	231
	23	97.45	81.89	1,923	90.56	79.75	1,186	98.25	94.18	1,824	96.80	96.57	228
	20	90.22	**82.67**	1,546	86.28	**79.99**	965	98.07	**94.44**	1,478	96.71	**96.59**	211
Clark	31	**99.31**	77.25	**3,116**	**95.66**	77.44	**1,670**	**98.91**	88.62	**2,855**	**99.68**	**99.42**	**251**
	27	99.07	79.37	2,796	93.90	78.29	1,522	98.90	92.26	2,554	99.67	**99.42**	241
	23	97.85	81.36	2,679	90.98	79.57	1,482	98.75	94.26	2,394	99.60	**99.42**	244
	20	88.60	**82.26**	2,567	83.35	**79.77**	1,456	97.73	**94.49**	2,306	99.43	99.41	239
Kraken-Q	31	**99.20**	76.84	6,224	**95.81**	**74.13**	5,308	**98.17**	87.46	7,023	**91.17**	**85.79**	3,809
	27	98.79	78.19	6,410	94.12	73.73	5,555	98.11	**89.89**	7,992	90.99	83.71	4,196
	23	96.67	**78.48**	7,015	90.57	72.35	6,329	97.21	89.07	8,989	90.46	79.27	4,574
	20	82.07	70.11	**9,437**	80.05	65.25	**9,537**	90.02	77.04	**10,961**	70.86	57.40	**5,819**
Clark-*E*	31	**99.55**	72.72	**32,450**	**98.11**	**74.58**	**28,988**	**99.00**	77.85	26,171	97.63	97.31	15,426
	27	99.43	74.67	29,897	96.93	75.68	28,459	98.93	84.86	**27,451**	97.47	97.18	**16,124**
	23	98.93	78.20	31,112	95.01	76.88	26,747	98.34	90.20	26,647	**98.56**	**98.32**	15,408
	20	94.74	**78.46**	30,029	90.57	76.60	25,789	96.61	**89.98**	26,545	93.94	93.82	15,587
Clark-*l*	27	98.45	62.30	1,525	92.11	69.64	861	95.96	52.00	1,705	99.49	98.94	143

Performance statistics for several choices of the *k*-mer length for NBC, KRAKEN, CLARK and their fast variants on the classification of “HiSeq”, “MiSeq”, “simBA-5” and “simHC.20.500” metagenomic datasets against the 695 genus-level targets; precision and sensitivity are expressed as percentages, while speed is expressed in 10^3^ reads per minute; KRAKEN-Q and CLARK-*E* are faster, but less accurate, variants of these tools; CLARK-*l* is a less memory-intensive version of CLARK which runs only for *k* = 27; experiments were carried out in single-threaded mode; ^∗^parameter *k* is referred as *N* in the NBC manuscript.

**Table 2:** **Species-level classification accuracy and speed of** CLARK, KRAKEN **, and** NBC **for four simulated metagenomes**

	**HiSeq**			**MiSeq**			**simBA-5**			**simHC.20.500**		
	***Prec***	***Sens***	***Speed***	***Prec***	***Sens***	***Speed***	***Prec***	***Sens***	***Speed***	***Prec***	***Sens***	***Speed***
NBC (*k*=15)	68.67	68.70	0.008	68.33	68.33	0.007	91.74	91.74	0.007	94.32	94.32	0.005
Clark (*k*=20)	69.44	61.46	272	70.72	62.45	239	91.32	82.48	269	94.34	94.32	96
Kraken (*k*=31)	74.00	53.49	2,332	77.72	58.72	1,361	92.99	78.70	1,976	84.67	84.31	237
Clark (*k*=31)	86.74	58.59	3,011	89.49	61.84	1,566	98.85	76.80	2,855	94.67	94.26	251
Kraken-Q (*k*=31)	75.88	50.78	6,224	78.07	53.68	5,308	92.67	74.39	7,023	82.40	74.84	3,809
Clark-*E* (*k*=31)	90.08	55.18	30,976	94.31	58.36	24,029	98.92	66.02	24,996	92.78	92.38	15,583
Clark-*l* (*k*=27)	85.35	53.95	1,676	85.89	64.91	904	85.55	46.28	1,702	94.06	93.53	141

Precision and sensitivity are expressed as percentages, while speed is expressed in 10^3^ reads per minute for NBC, KRAKEN, and CLARK on the classification of “HiSeq”, “MiSeq”, “simBA-5” and “simHC.20.500” metagenome datasets against the 1473 species-level targets, in single-threaded mode.

For a given level in the taxonomy tree (e.g., genus), we define *precision* as the fraction of correct assignments over the total number of assignments, and *sensitivity* as the ratio between the number of correct assignments and the number of objects to be classified. In order to have a fair comparison against KRAKEN’s assignments, when KRAKEN produces an assignment that is not available at or below the genus or species level, it is then considered as not assigned.

**Figure 1:**
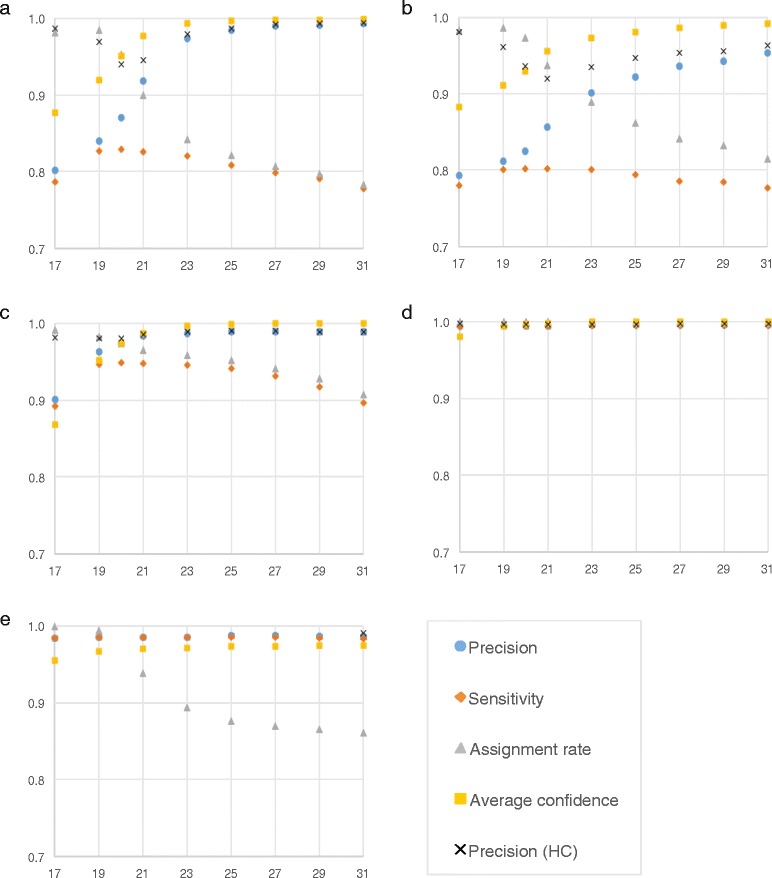
Classification performance of CLARK for several *k*-mer length and for various datasets.CLARK’s precision, sensitivity, assignment rate, average confidence scores and precision of high confidence assignments (HC) for several choices of the *k*-mer length on the “HiSeq” metagenomic dataset **(a)**, the “MiSeq” metagenomic dataset **(b)**, the “simBA-5” metagenomic dataset **(c)**, the “simHC.20.500” metagenomic dataset **(d)**, and barley unigenes **(e)**. **(a)** – **(d)** are results of the classification against the 695 genus-level targets.

Table 1 reports precision, sensitivity and processing speeds (in 10^3^ reads per minute) obtained by NBC, KRAKEN and CLARK on the HiSeq, MiSeq, simBA-5 and simHC.20.500 datasets, for several values of the *k*-mer length. The table illustrates how the performance of these tools is affected by the choice of *k*. By increasing *k* one generally increases precision, but can lower sensitivity (also see Figure 1). To carry out a fair comparison between tools, we decided to first determine NBC’s and KRAKEN’s optimal *k*-mer length, and then run CLARK with a value of *k* that would match either sensitivity or precision.

NBC was tested with *k*=11,13,15. We observed that *k*=15 produced the highest sensitivity on all datasets. The value *k*=15 is the highest possible value, which is recommended by the authors of [8] for datasets composed of short reads. Since NBC produces detailed statistics on the assignments, we executed CLARK in “full” mode for a fair comparison. Using *k*=20 for CLARK (full mode) we obtained a similar sensitivity to NBC (CLARK is actually more sensitive than NBC on HiSeq and simHC.20.500). At the same level of sensitivity of NBC, CLARK achieves a higher precision and it is thousands of times faster.

In the case of KRAKEN, *k*=31 was the value used in [11] for HiSeq, MiSeq and simBA-5 and it is supposed to achieve the highest precision. Nonetheless, we tried to run KRAKEN for other values of *k*. As expected, Table 1 shows that *k*=31 produces the best precision for all the datasets. For this comparison, we also ran CLARK with *k*=31. Observe that CLARK (default mode) is slightly less sensitive than KRAKEN but is more precise and faster. The difference in speed is significant for all datasets of short reads (300−800 thousand additional reads/min). On simHC.20.500, KRAKEN and CLARK achieve the same speed due to the fact that these datasets contain longer reads. Finally, CLARK has better sensitivity than KRAKEN on simHC.20.500.

The same comparisons were carried out between the two variants of KRAKEN and CLARK optimized for speed, called KRAKEN-Q and CLARK-*E* (*E* for “Express”, see Methods section). As indicated in Table 1, KRAKEN-Q achieves the best precision for all the datasets when *k*=31, which is consistent with [11]. However, when *k*=31CLARK-*E* runs four–five times faster than KRAKEN-Q and is also more precise. In addition, observe that as we decrease *k*, both variants gets faster but CLARK-*E* maintains a precision above 90% while KRAKEN-Q produces progressively lower precisions.

In the last row of Table 1, we report the performance of CLARK-*l*, another variant of CLARK designed for low RAM architectures that runs only for *k*=27 (see Methods section). CLARK-*l* performs assignments with a lower precision than CLARK (the difference is at most 3.5% in these experiments) but can process more than 1.5 million of reads per minute on HiSeq or simBA-5, and only uses about 4% of the memory used by CLARK (see Additional file 1: Table S1).

All experimental results reported so far were obtained in single-threaded mode. If a multi-core architecture is available, CLARK and KRAKEN can take advantage of it. In Additional file 1: Table S2, we summarize the classification speed of the two tools using 1, 2, 4 or 8 threads for *k*=31. Observe that using eight threads, CLARK achieves a speed-up of 5.2x compared to one thread, while KRAKEN only achieves a speed-up of 1.2x. When comparing CLARK-*E* to KRAKEN-Q, we can make similar observations. In general, note that CLARK-*E* is at least five times faster than KRAKEN-Q, independently of the number of threads used.

For the analysis at the species level, we repeated the classification of the objects in the four datasets described above against species-level targets. This time we used values of *k* that allowed best sensitivity for NBC (*k*=15) and best precision for KRAKEN (*k*=31). Observe in Table 2 that NBC achieves the best sensitivity on all datasets. However, when CLARK is ran in full mode using *k*=20, it achieves a higher precision than NBC on HiSeq, MiSeq and simHC.20.500, and is several orders of magnitude faster. In addition, CLARK in default mode using *k*=31 achieves higher precision than KRAKEN on all datasets (as much as 10% higher on HiSeq and MiSeq) when *k*=31. CLARK also outperforms the speed of KRAKEN on HiSeq, MiSeq and simBA-5. On simHC.20.500, since the reads are much longer, the speed of KRAKEN and CLARK are comparable. But, CLARK has higher sensitivity than KRAKEN on HiSeq, MiSeq and simHC.20.500. Finally, the fast variant CLARK-*E*, as previously observed for the experiments at the genus level, outperforms KRAKEN-*Q* in both speed and precision.

#### Human microbiome samples

In the second experiment, we used CLARK to classify Human Microbiome Project reads against 695 genus-level targets described above. This time, however, the “ground truth” was not available.

Using *k*=31, CLARK was able to assign 42.1% of the reads in SRS015072 (mid-vagina), 30.8% of the reads in SRS019120 (saliva) and 49.8% of the reads in SRS023847 (nose). KRAKEN achieved similar rates of assigned reads using *k*=31. Reducing *k* would increase the number of assignments, at the cost of increasing the probability of misclassification. We investigated whether we could take advantage of CLARK’s confidence scores to compensate for a smaller value of *k*, and improve the fraction of assigned reads.

Figure 1a to Figure 1d show that CLARK’s sensitivity on the four datasets is the highest for *k*=20 or *k*=21. However, the precision for *k*=20 and *k*=21 is about 15% lower than for *k*=31, which implies that a large proportion of assignments may be incorrect. We have strong experimental evidence that shows that the higher is CLARK’s confidence score for an assignment, the more likely that assignment is correct (see Additional file 1: Supplementary Note 2). In addition, we observe in Figure 1a to Figure 1d that the precision of high confidence assignments is higher than the average precision of all assignments, and is relatively constant for all *k*-mer length. The idea is to use *k*=20 to maximize the number of assigned reads, but only consider high confidence assignments to increase the precision. We call an assignment *high confidence* if the confidence score is higher than 0.75, *low confidence* otherwise.

Observe in Table 3 that the number of high confidence assignments for *k*=20 is significantly higher than for *k*=31. The relative increase in assignments is about 40% (from 42.1% to 62.3% in SRS015072, 30.8% to 55.1% on SRS019120, and 49.8% to 68.3% on SRS023847). Table 3 also reports the most frequent five genera in high confidence assignments. For the saliva sample, the dominance of *Streptococcus*, *Haemophilus* and *Prevotella* is consistent with findings in [2] and [11]. Study [21], which focused on salivary microbiota of 35 inflammatory bowel disease patients, also reports *Streptococcus*, *Prevotella*, *Neisseria*, *Haemophilus* and *Veillonella* as dominant genera. Concerning the mid-vagina sample, we have found that *Lactobacillus* is the dominant genus, in agreement with findings reported in [2,22,23]. The proportion of *Lactobacillus* we have identified (64.7%) is very close to the reported proportion (69%–71%) in [22,23]. The presence of *Pseudomonas* and *Gardnerella* is expected because some individuals who lack *Lactobacillus* have instead *Gardnerella* or *Pseudomonas* as the predominant bacteria [22,23]. In the nose sample, the high presence of *Propionibacterium* and *Staphylococcus* is consistent with the results in [2].

**Table 3:** **Summary of the Genus-level classification for three Human Microbiome Project datasets (** ***k*** **=20)**

***SRS ID***	***High confidence***	***Low confidence***	***No assignment***	***Average***	***Most frequent genera (high***
	***assignments (%)***	***assignments (%)***	***(%)***	***confidence score***	***confidence assignments)***
015072	62.3%	25.9%	11.8%	0.868	*Lactobacillus* (64.7%)
(vagina)					*Pseudomonas* (7.3%)
					*Desulfosporosinus* (4.4%)
					*Clostridium* (1.7%)
					*Gardnerella* (1.2%)
019120	55.1%	28.2%	16.7%	0.842	*Streptococcus* (27.2%)
(mouth)					*Haemophilus* (15.0%)
					*Prevotella* (11.4%)
					*Neisseria* (5.0%)
					*Veillonella* (2.9%)
023847	68.3%	23.8%	7.9%	0.954	*Propionibacterium* (61.5%)
(nose)					*Staphylococcus* (8.5%)
					*Achromobacter* (7.5%)
					*Alteromonas* (6.3%)
					*Desulfosporosinus* (5.0%)

Columns: (1) short read sample ID; (2) percentage of high confidence assignments; (3) percentage of low confidence assignments; (4) percentage of unassigned reads; (5) average confidence score for all assignments; (6) five most frequent genera in high confidence assignments (listed in decreasing order). An assignment is *high confidence* if the confidence score is higher than 0.75, *low confidence* otherwise.

### Classification of barley BACs and unigenes to chromosome arms and centromeres

Inputs to this classification task were (1) barley chromosome arms (targets) and (2) barley BACs or unigenes (objects). Samples of each barley chromosome arm were obtained using flow-sorting [24]. The procedure to obtain gene-rich barley BACs was described in [25]. Sequences for chromosome arms and BACs were generated on an Illumina HiSeq 2000 instrument by J. Weger at UC Riverside.

For the targets, we processed thirteen datasets of shotgun sequenced reads: one for barley chromosome 1H and twelve for barley chromosome arms (namely, 2HL, 2HS, 3HL, 3HS, 4HL, 4HS, 5HL, 5HS, 6HL, 6HS, 7HL, and 7HS). After quality-trimming the reads, we had a total of about 181 Gbp of sequence data. The cumulative size of the assembled barley chromosome arms obtained via SOAPDENOVO [26] resulted in about 2 Gbp (about 40% of the barley genome).

The objects were 50,938 barley unigenes (transcript assembly from ESTs) obtained from [27] for a total of about 222.4 Mbp. Additionally, we trimmed short reads for 15,721 BACs obtained from [25], for a total of about 1.73 Gbp. We also had access to 15,697 BAC assemblies (not all BACs had a sufficient number of reads for an assembly) for a total of about 1.80 Gbp. While the genomic location for the majority of these “objects” was unknown, we had 1,652 unigenes for which a location was derived from the Golden Gate oligonucleotide pool assay (OPA) [28], which allowed us to determine a presumed location of 2,252 BACs [25]. We should point out that although we have used these locations as the “ground truth” to establish the accuracy of the classification, our observations indicate about 5% errors in these OPA assignments [25].

As stated above, the most critical parameter in CLARK is the length of the *k*-mer used for classification. By assuming that the subset of the unigenes that have a location via OPA are correct, we were able to estimate CLARK’s precision and sensitivity for various choices of *k*. Figure 1e shows these statistics, along with the assignment rate (fraction of unigenes assigned) and the average confidence score for all assignments. Observe that as *k* increases, the number of assignments decreases but the precision/sensitivity increases. Based on this analysis we determined that *k*=19 represents a good tradeoff for this dataset.

Table 4 summarizes CLARK’s assignment of barley unigenes (assemblies) to barley chromosomes arms (assemblies) using *k*=19. When both targets and objects are assemblies, we call this an “A2A” assignment. Observe that most of the assignments have high confidence and they are relatively evenly distributed among barley chromosome arms (the seven barley chromosomes are believed to be relatively similar in length). Observe in Figure 1e that CLARK’s precision and sensitivity for this classification task is very high (both at 98.49%) while the average confidence score is above 0.96, and 99.44% of unigenes are assigned.

**Table 4:** **Summary of** CLARK **’s assignment of 50,646 unigenes (EST assemblies) to barley chromosome arms (assemblies) and centromeres (** ***k*** **=19)**

***Targets***	***19-mers***	***Discriminative 19-mers***	***Assignments***	***Low confidence***	***High confidence***
1H	180,176,713	108,894,740	8,197	21.1%	78.9%
2HC	-	814,357	15	93.3%	6.7%
2HL	103,679,920	64,700,161	4,776	15.8%	84.2%
2HS	90,912,314	54,449,430	3,334	17.3%	82.7%
3HC	-	1,532,968	29	79.3%	20.7%
3HL	123,140,951	78,158,244	4,726	16.7%	83.3%
3HS	111,951,787	70,473,478	3,159	20.4%	79.6%
4HC	-	3,105,047	54	50.0%	50.0%
4HL	106,999,773	64,749,958	3,531	14.4%	85.6%
4HS	89,027,872	51,612,790	2,468	16.4%	83.6%
5HC	-	604,030	9	88.9%	11.1%
5HL	117,915,094	77,128,375	6,111	12.2%	87.8%
5HS	58,067,400	34,037,607	1,619	17.8%	82.2%
6HC	-	469,530	9	100.0%	0.0%
6HL	74,485,223	44,221,184	2,973	12.4%	87.6%
6HS	111,834,123	83,957,421	2,721	24.4%	75.6%
7HC	-	795,923	9	88.9%	11.1%
7HL	92,603,503	58,159,248	3,556	10.9%	89.1%
7HS	90,217,777	55,276,671	3,350	12.6%	87.4%
*Total*	1,351,012,450	853,141,162	50,646	16.5%	83.5%

Columns: (1) barley chromosome 1H, twelve chromosome arms, and six centromeres; (2) number of distinct *k*-mers in each target; (3) number of discriminative *k*-mers present in target sequences (must occur at least once); (4) number of assigned objects per target; (5) number of low confidence assignment per target; (6) number of high confidence assignment per target; (7) percentage of low confidence assignment (as a fraction of the total number of assigned objects per target); (8) percentage of high confidence assignment (as a fraction of the total number of assigned objects per target).

Additional file 1: Table S3 presents a summary of CLARK’s assignment of barley BACs (assemblies) to arms (assemblies), while Table 5 refers to the same assignments based on the reads instead of the assemblies (“R2R” assignment). The consistency between these results (same distribution of BACs assignments over chromosome arms, and similar proportion of high and low confidence assignments) demonstrates the robustness of our approach. The agreement with OPA-based locations is 92.9% for R2R assignments, and 93.2% for A2A assignments. Observe that the agreement for BAC/arm assignments is lower than unigene/arm assignments (98.49%).

**Table 5:** **Summary of** CLARK **’s assignment of 15,665 BACs (represented as reads) to barley chromosome arms (reads) and centromeres (** ***k*** **=19)**

***Targets***	***19-mers***	***Discriminative 19-mers***	***Assignments***	***Low confidence***	***High confidence***
1H	448,768,897	126,997,864	2,068	4.2%	95.8%
2HC	-	1,738,722	0	-	-
2HL	451,729,142	102,959,160	1,417	2.1%	97.9%
2HS	401,605,473	79,225,936	1,071	2.4%	97.6%
3HC	-	4,631,639	0	-	-
3HL	553,420,081	138,939,217	1,423	2.2%	97.8%
3HS	538,777,930	113,354,224	892	3.5%	96.5%
4HC	-	6,428,726	70	14.3	85.7%
4HL	494,923,209	106,930,230	1,127	2.3%	97.7%
4HS	462,144,322	85,650,765	888	3.4%	96.6%
5HC	-	1,643,194	0	-	-
5HL	558,710,983	121,491,586	1,657	2.3%	97.7%
5HS	281,062,766	57,181,745	658	2.4%	97.6%
6HC	-	1,287,133	0	-	-
6HL	311,443,157	70,856,097	1,136	2.0%	98.0%
6HS	877,169,677	255,819,549	850	2.9%	97.1%
7HC	-	1,697,991	0	-	-
7HL	366,612,780	82,987,499	1,175	2.0%	98.0%
7HS	365,475,556	83,848,867	1,233	2.8%	97.2%
*Total*	6,111,843,973	1,443,670,144	15,665	2.7%	97.3%

Columns: (1) barley chromosome 1H, twelve chromosome arms, and six centromeres; (2) number of distinct *k*-mers in each target; (3) number of discriminative *k*-mers present in target sequences (must occur at least twice); (4) number of assigned objects per target; (5) number of low confidence assignment per target; (6) number of high confidence assignment per target; (7) percentage of low confidence assignment (as a fraction of the total number of assigned objects per target); (8) percentage of high confidence assignment (as a fraction of the total number of assigned objects per target).

Finally, we compared CLARK against (1) the BLAST-based method used in [25] for BAC-arm assignment (A2A); and (2) the assignments provided in [16,29]. For (1), CLARK assigned 13,706 BACs (of which 2,252 have a prior OPA-based location) while the BLAST-based method assigned 13,583 BACs (of which 2,238 have a prior OPA-based location). CLARK’s precision and sensitivity were 93.2% and 93.2%, respectively, while BLAST-based’s precision and sensitivity were 92.4% and 91.9%, respectively. BLAST-based and CLARK disagreed on 19 assignments; within these 19 disagreements, CLARK agreed with the GoldenGate assays on seven cases, and BLAST-based agreed on four cases. In (2), we examined the assignment for the 1,037 BACs that were sequenced by our group and by Leibniz-Institut fur Pflanzengenetik und Kulturpflanzenforschung, Gatersleben, Germany (IPK) [16] and we identified only 42 disagreements (4% of the total); among these disagreements, 19 had an independent assignment via POP-seq [29]. In 15 cases out of 19, our assignment agreed with the POP-seq assignment. For the 23 disagreements for which there was no POP-seq assignment, we compared the assembled BACs and we discovered 6 cases in which the sequences were less than 30% similar, suggesting a naming error. In summary, there were only a handful of cases where the disagreement could not be readily explained.

### Performance dependency on the *k*-mer length

To determine the optimal value of *k* for a particular dataset one can take advantage of prior knowledge, as we did in the case of unigene/BAC assignment to chromosomes. In that case, we had 1,657 unigenes for which the correct BAC assignment (approximately 95% accuracy) was experimentally determined via Illumina GoldenGate assay (BOPA1 and BOPA2). Given these known assignments, we estimated precision and sensitivity, as well as the average confidence score for all assignments and the assignment rate (see Figure 1e). Observe that *k*=19 maximizes all four measurements. Higher precision and average confidence score can be achieved by using higher *k* but at the cost of decreasing sensitivity and assignment rate.

Similar evaluation were carried out on the metagenomic datasets. Figure 1a to Figure 1d show precision, sensitivity, as well as assignment rate and average confidence score as a function of *k*. In both cases we observe that as we increase *k*, precision and the average confidence score are increasing, while the sensitivity is decreasing. We observe that the maximum sensitivity is achieved for *k* in the range 19–22 for all metagenomic datasets, independently of the reads length or complexity.

As a consequence, for high sensitivity (or high number of assignments) one must choose *k* between 19 and 22. For high precision (or high confidence score) one must choose *k* higher than 26. The current implementation supports *k* up to 32.

## Conclusions

We have presented CLARK, a new method for metagenomic sequence classification and chromosome/arm assignments of DNA sequences.

Experimental results demonstrate that CLARK has several advantages over alternative methods. (i) CLARK is able to classify short metagenomic reads with high accuracy at multiple taxonomic ranks (i.e., species and genus level) and its assignments on real metagenomic samples are consistent with findings published in the literature. (ii) CLARK can achieve the same or better accuracy than the state-of-the-art metagenomic classifiers. (iii) The classification speed of CLARK, in the context of metagenomics, is unmatched. CLARK can classify 32 million metagenomic short reads per minute, which is five times faster than KRAKEN. In addition, CLARK “scales” better on a multi-core architectures: the speed-up one can obtain by adding more threads is higher than KRAKEN. (iv) CLARK is able to output confidence scores, is user-friendly and self-contained (unlike most of other classifiers, it does not require external tool such as BLAST or MEGABLAST, etc). (v) CLARK can be executed with relatively small amounts of RAM (unlike LMAT) or disk space (unlike PHYMMBL or KRAKEN). Indeed, LMAT can use about 500 GB of RAM, while the maximum amount of RAM needed by CLARK is less than 165 GB (see Additional file 1: Table S1). PHYMMBL or KRAKEN require respectively about 120 GB and 140 GB of disk space to run, while CLARK requires 40–42 GB for classification. (vi) In the context of genomics, CLARK can classify BACs and transcripts with better accuracy than previously used BLAST-based method [25], and can infer centromeric regions.

Even though in this manuscript we focus the attention on genus and species level classification, CLARK is expected to work also at higher taxonomic levels such as phylum, family or class. As it is now, however, CLARK cannot take advantage of taxonomic tree structures. We believe that CLARK will be useful in a variety of applications in metagenomics and genomics. For instance, we have used CLARK to identify chimerism and vector contamination in sequenced BACs.

## Methods

### Building target-specific *k*-mer sets

CLARK accepts inputs in fasta/fastq format; alternatively the input can be given as a text file containing the *k*-mer distribution (i.e., each line contains a *k*-mer and its number of occurrences). CLARK first builds an index from the target sequences, unless one already exists for the specified input files. If a user wants to classify objects at the genus level (or another taxonomic rank), he/she is expected to generate targets by grouping genomes of the same genus (or with the same taxonomic label). This strategy represents a major difference with other tools (such as LMAT, or KRAKEN). The index is a hash-table storing, for each distinct *k*-mer *w* (1) the ID for the target containing *w*, (2) the number of distinct targets containing *w*, and (3) the number of occurrences of *w* in all the targets. This hash-table uses separate chaining to resolve collisions (at each bucket). CLARK then removes any *k*-mer that appears in more than one target, except in the case of chromosome arm assignment. In the latter case, *k*-mers shared by the two arms of the same chromosome are used to define centromeric regions of overlap. Also, *k*-mers in the index may be removed based on their number of occurrences if the user has specified a minimum number of occurrences. These rare *k*-mers tend to be spurious from sequencing errors. Other metagenomic classifiers like KRAKEN and LMAT do not offer this protection against noise, which is very useful when target sequences are reads (or low-quality assemblies). Then, the resulting sets of target-specific *k*-mers are stored in disk for the next phase. The time and memory needed to create the index (for *k*=31) are given in Additional file 1: Table S1. This table also contains the time and memory required by NBC and KRAKEN. Observe that CLARK is faster than NBC and KRAKEN to create the index, and it uses less RAM and disk space than KRAKEN for classifying objects.

The concept of “target-specific *k*-mers” is similar to the notion of “clade-specific marker genes” proposed in [7] or “genome-specific markers” recently proposed in [30]. While CLARK uses exact matching to identify the target-specific *k*-mers derived from any region in the genome, the authors in [7] disregard intergenic regions. The authors of [30] focus on strain-specific markers identified by approximate string matching, while CLARK uses exact matching. Another important difference is that the method presented in [30] relies on MEGABLAST [31] to perform the classification, which is several orders of magnitude slower than KRAKEN [11].

For users that want to run CLARK on workstations with limited amounts of RAM, we have designed CLARK-*l* (“light”). CLARK-*l* is a variant of CLARK that has a much smaller RAM footprint but can classify objects with similar speed and accuracy. The reduction in RAM can be achieved by constructing a hash-table of smaller size and by constructing smaller sets of discriminative *k*-mers. Instead of considering all *k*-mers in a target, CLARK-*l* samples a fraction of them. CLARK-*l* uses 27-mers (27-mers appeared to be a good tradeoff between speed, low memory usage and precision) and skips four consecutive/non-overlapping 27-mers. As a result, CLARK-*l*’s peak RAM usage is about 3.8 GB during the index creation, and 2.8 GB when computing the classification (see Additional file 1: Table S1). CLARK-*l* has also the advantage to be very fast in building the hash table. Table 1 includes the performance of CLARK-*l*. While the precision and sensitivity are lower compared to CLARK, CLARK-*l* still achieves high precision and high speed.

### Sequence classification

In the full mode, once the index containing target-specific *k*-mers has been created, CLARK creates a “dictionary” that associates *k*-mers to targets. Then, CLARK iteratively processes each object: for each object sequence *o*CLARK queries the index to fetch the set of *k*-mers in *o*. A “hit” is obtained when a *k*-mer (either forward or reverse complement) matches a target-specific *k*-mer set. Object *o* is assigned to the target that has the highest number of hits (see algorithmic details in Additional file 1: Supplementary Note 1 and Additional file 1: Table S5). The confidence score is computed as *h*_1_/(*h*_1_+*h*_2_), where *h*_1_ is the number of hits for the highest target, and *h*_2_ is the number of hits for the second-highest target.

The rationale to remove common *k*-mers between targets (at any taxonomy level defined by the user) is that they increase the “noise” in the classification process. If they were present, more targets could obtain the same number of hits which would complicate the assignment. If such conflicts can be avoided, then there is no need to query the taxonomy tree, and find, for example, the lowest common ancestor taxons for “conflicting nodes” to resolve them as it is done in other tools (e.g., KRAKEN or LMAT). Observe in Additional file 1: Figure S1, that most of CLARK’s assignments have high confidence scores. Observe that at least 95% of all assignments in HiSeq, MiSeq, simBA-5 and simHC.20.500 made by CLARK in the full mode, have confidence scores equal to 1 (i.e., exactly one target gets hits), and the average confidence scores in all these assignments is 0.997. This implies that, on average, the number of hits for the top target (which will receive the assignment) is about 336 times higher than the second. Thus, CLARK, unlike LMAT or KRAKEN, does not need the taxonomy tree to classify objects, instead one “flat” level is clearly sufficient.

If users are not interested in collecting confidence scores and all hit counts, then it is recommended to use the default mode of CLARK. In this mode, CLARK stops querying *k*-mers for an object as soon as there is at least one target that collects at least half of the total possible hits. Also, this mode loads in main memory about half of the target-specific *k*-mers. This is done by alternatively loading or skipping target-specific *k*-mers based on their index positions. CLARK runs significantly faster in default mode (2–5 times faster in our experiments) with negligible degradation of sensitivity and assignment rate. Also, the RAM usage is significantly lower than the full mode (up to 50% lower in our experiments). If speed is the primary concern, we have designed an “express” variant of CLARK called CLARK-*E*. CLARK-*E* is based upon Theorem 1 (see Additional file 1: Supplementary Note 1), which states that if an object originates from one of the targets then either one or no target will be hit from the *k*-mers in the object. Since we use target-specific *k*-mer sets, at most one target can be associated to the *k*-mers of an object. In addition, we reduce the number of queries to the database by considering a sample of the *k*-mers in the object. So CLARK-*E* only queries non-overlapping *k*-mers, and the object is assigned to the first target that obtains a hit. This optimization allows CLARK-*E* to be extremely fast compared to CLARK/KRAKEN (see Table 1), while maintaining high precision and sensitivity.

### Running time analysis

All experiments presented in this study were run on a Dell PowerEdge T710 server (dual Intel Xeon X5660 2.8 Ghz, 12 cores, 192 GB of RAM). CLARK-*l* was also run on a Mac OS X, Version 10.9.5 (2.53 GHz Intel Core 2 Duo, 4 GB of RAM). When comparing KRAKEN to CLARK in their default mode, and KRAKEN-Q to CLARK-*E*, we always set KRAKEN to “preload” its database in main memory and print results to a file (instead of the standard output) to achieve the highest speed. For consistency, CLARK was also run under the same conditions. For the results in Table 1 and Table 2, CLARK (v1.0), NBC (v1.1), and KRAKEN (v0.10.4-beta) were run in single-threaded mode, three times on the same inputs in order to smooth fluctuations due to I/O and cache issues (the reported numbers are best values). We have also run the latest version of Kraken (v0.10.5-beta) and we did not observe a significant variation of accuracy and usage of RAM. However, we observed a 15% decrease in the classification speed compared to version v0.10.4-beta. The software tool CLARK is available for download at http://clark.cs.ucr.edu/.

## Ethics statement

All human data used in this study are from the Human Microbiome Project [2,3], which is a free and publicly available database.

## Additional file

Additional file 1 **Supplementary Material.** Detail about the mathematical modeling, the impact of the *k*-mer length on results, the analysis of the confidence scores, and the software implementation.
