# Supplementary material for: CLARK: fast and accurate classification of metagenomic and genomic sequences using discriminative k-mers
Source: BMC Genomics. 2015 Mar 25;16(1):236. doi: 10.1186/s12864-015-1419-2 (PMC4428112; doi:10.1186/s12864-015-1419-2)
Supplement: Supplementary file 1 — Supplementary Material. Detail about the mathematical modeling, the impact of the k-mer length on results, the analysis of the confidence scores, and the software implementation. [file 12864_2015_1419_MOESM1_ESM.pdf]

CLARK: Fast and Accurate Classification of Metagenomic and Genomic Sequences using Discriminative k-mers

Supplementary Material

Supplementary Note 1: Algorithmic details

Notations and Problem Definition

Object and targets are described by their *sequence* which is a non-empty string over the alphabet $\Sigma = \{A, T, G, C\}$ of *nucleotides* (U can replace T in the case of mRNA). Observe that two bits are sufficient to identify a nucleotide. Given a sequence s, we use $|s|$ to denote its length. A *target* is a sequence representing either a chromosome arm, or a chromosome, or a genome, or a species (i.e., a set of genomes from different individuals), or a genus (i.e., a set of genomes). We use the variable n to indicate the number of targets. An *object* is a sequence that is assumed to originate from at most one of the n targets. We use the variable p to indicate the number of objects.

We say that a non-empty object s *originates* from target g if sequence s is a substring of sequence g. Given n targets $\{g_1, g_2, \ldots, g_n\}$ we say that a sequence s is *specific* to target g_c (or g_c-*specific*) $1 \leq c \leq n$, if s is a substring of g_c and s is not a substring of any other target. We say that a sequence s is a *repeat* of $\{g_1, g_2, \ldots, g_n\}$ if it is a substring of more than one target.

Given a positive integer k, a k-*mer* is any sequence of k consecutive nucleotides. Given that $|\Sigma| = 4$, there is a total of 4^k possible k-mers, i.e., any k-mer can be then associated to a unique *dimension* ranging from 1 to 4^k. It is easy to observe that exactly $N - k + 1$ k-mers (distinct or not) can be extracted from a sequence of length N, when $k \leq N$.

The assignment problem can be defined as follows. Given a set of targets$\{g_1, g_2, \ldots, g_n\}$, a set of objects $\{s_1, s_2, \ldots, s_p\}$, and a positive integer k, assign each object s_i to the target g_{c^*}, such that the number of g_c-specific k-mers contained for s_i is the highest (where ties are broken arbitrarily) for $c = c^*$, where $1 \leq c \leq n$.

The k-*spectrum* $T(s)$ of an object s is the vector of size 4^k defined as follows: for any $1 \leq i \leq 4^k$, $T(s)_i$ is the number of occurrences in s of the k-mer with dimension i. Now consider $(E_k, ||.||_1)$, where $E_k = \mathbb{R}^{4^k}$ is a normed vector space of dimension 4^k and $||.||_1$ is the 1-norm. Although spectrums are vectors of integers, it is more convenient to consider the set $\mathbb{R}$ rather than $\mathbb{Z}$ because the former is a field. Thus, $(E_k, < \cdot|\cdot >)$, where $< \cdot|\cdot >$ is the standard dot product, is an Euclidean space, on which useful notions such as projection and orthogonality can be defined. If $\vec{e_i}$ is the *unit vector* (entry i equal to 1 and 0 everywhere else), then $(\vec{e}_1, \vec{e}_2, \ldots, \vec{e}_{4^k})$ is the *canonical basis* of E_k.

The 1-norm of a vector $\vec{v} \in E_k$ is defined as $||\vec{v}||_1 = \sum_{i=1}^{4^k} |v_i|$. Since E_k is a vector space of finite dimensions, all p-norms are equivalent in E_k. However, we prefer the 1-norm due some of its properties. For instance, for any k-spectrum $T(s)$ for a sequence s of length N, we have $||T(s)||_1 = N - k + 1$. In other words, sequences of same length have the same 1-norm.

Probability of Two Sequences to Share the Same k-spectrum

We first observe that the mapping between a sequence and its spectrum is not one-to-one (i.e., it is not invertible), because the spectrum ignores the order of k-mers in the sequence. The consequence is that two or more distinct sequences can have the same spectrum. For example, the spectrums of all $N - 1$ circular rotations of a string of length N are identical to each other.

We now proceed to compute the probability that a pair of sequences (targets or objects) of length N share

the same k-spectrum. The problem of recovering a sequence from a set of k-mers is one of the "flavors" of genome assembly. From the k-spectrum one can build the corresponding *de Bruijn graph* (nodes are k-mers and edges connect two nodes if the two corresponding k-mers have a $k-1$ overlap). Any Eulerian path of this graph recovers one of sequences having such spectrum [1]. Here we want to count the number of sequences with the same k-spectrum, which is equal to the number of distinct Eulerian paths in the corresponding de Bruijn graph. Given a sequence s, we call $B_{k,N}$ the set of distinct sequences of length N whose k-spectrum is $T(s)$. Then, $|B_{k,N}|$ is the number of Eulerian paths in the de Bruijin graph G_s built from the k-spectrum of s.

Let us consider a set D of sequences of length N and an integer k. Let s, s' be two sequences in D. The probability that s and s' have the same k-spectrum is

$$P\left(T(s) = T(s')|s \neq s'\right) = \frac{|B_{k,N} \cap D|}{|D| - 1}$$

Since we will be using spectrums for classification, we want this probability of a conflict to be as small as possible. However, $|B_{k,N} \cap D|$ is not easy to evaluate for a generic set D of sequences. We can compute this quantity when D is the set of all sequences of length N. In this case, $|D| = 4^N$ and $|B_{k,N} \cap D| = |B_{k,N}|$.

The quantity $|B_{k,N}|$ is an upper bound to the number of Eulerian paths in G_s for a sequence s of length N. Thus, we have $|B_{k,N}| \leq (N-k+1)4^{N-k-3} \cdot 3 \cdot 2 \cdot 1$, because there are at most $(N-k+1)$ possibilities for choosing the first k-mer, then at most four distinct k-mers for the second position, then at most four distinct k-mers for the third position, and so on and so forth. For the last three positions there are three, then two, and one k-mer. Thus,

$$P\left(T(s) = T(s')|s \neq s'\right) \leq \frac{(N-k+1)4^{N-k-3} \cdot 3 \cdot 2 \cdot 1}{4^N - 1} = \frac{2(N-k+1)}{4^{k+2}} \tag{1}$$

For instance, when N is small (say, $N = 1000$), and k=12, we can estimate that $P(T(s) = T(s')|s \neq s') \leq 10^{-5}$. If N is bigger (say, $N = 10^8$, which the size of a small genome) and k = 12, then $P(T(s) = T(s')|s \neq s') \leq 0.7451$. For $N = 10^8$ and k = 19 we get $P(T(s) = T(s')|s \neq s') \leq 4.547\ 10^{-5}$, and for $N = 10^9$ and k = 19, we get $P(T(s) = T(s')|s \neq s') \leq 4.547\ 10^{-4}$.

Inequality 1 can be used to determine the value of k that will make the probability of a spectrum conflict small enough (given N). Recall that we assumed that D contains all possible sequences of length N. When $|D| << 4^N$, it is reasonable to assume that Inequality 1 still holds when k is large enough, since in this case $|B_{k,N} \cap D| = 0$ (e.g., consider the extreme case $N = k$).

Spectral decomposition

Now we describe how k-spectrums can be used to assign objects to targets. Given a target g_c, $1 \leq c \leq n$, let $T(g_c)$ be its k-spectrum. Henceforth, we assume that vectors $T(g_1), T(g_2), T(g_3), \ldots, T(g_n)$ are non-null and linearly independent, i.e., the determinant of the matrix obtained from these vector is not zero:

$$det\left[T(g_1), T(g_2), T(g_3), \ldots, T(g_n)\right] \neq 0 \tag{2}$$

This assumption is met in practice and it is sufficiently general due to the fact that sequences from distinct targets contain unique substrings. From Inequality 1, we can also choose k large enough so the probability of two distinct sequences to share the same spectrum to be as small as needed.

Let B_c be the basis of unit vectors such that these unit vectors are associated to non-zero-count dimensions in the k-spectrum of $T(g_c)$, i.e., $B_c = (\vec{e_i})_{i \in I_c}$, where $I_c = \{i,\ i \in \{1, 2, 3, \dots, 4^k\} \mid T(g_c).\vec{e_i} \neq 0\}$. Since B_c contains all non-null dimensions from $T(g_c)$, we can define $E_k^c = span(B_c)$, which is the space described by linear combinations of the unit vectors in B_c. E_k^c represents the vector space associated to the k-spectrum of target g_c.

Now we are going to build another basis, but only for target-specific k-mers. Let $\widetilde{B}_c$ be the basis of unit vectors corresponding to the set of dimension of non-zero counts in the k-spectrum of $T(g_c)$, which has at the same time, zero counts in the spectrum of other targets, i.e., $\widetilde{B}_c = (\vec{e_i})_{i \in \widetilde{I}_c}$, where $\widetilde{I}_c = \{i,\ i \in \{1, 2, 3, \dots, 4^k\} \mid T(g_c).\vec{e_i} \neq 0$ and for all $c' \neq c$ we have $T(g_{c'}).\vec{e_i} = 0\}$. By the Equation 2, we have for all $c,\ \widetilde{B}_c \neq \emptyset$ (if for some $c,\ \widetilde{B}_c = \emptyset$, then we need to increase k). Therefore, we can define $\widetilde{E}_k^c = span(\widetilde{B}_c)$, which is the vector space built from all subspaces specific to E_k^c.

$\widetilde{E}_k^c$ is called *target-specific k-mer space* of g_c or simply g_c-specific k-mer space. If the set of targets $\{g_1, g_2, \dots, g_n\}$ represents chromosome arms, and the sequences for the two arms overlap each other, then it is possible to define centromere-specific k-mer spaces. Given (c, c') representing the two arms of the same chromosome (for example in barley, 2HS and 2HL), let $\widetilde{B}_{c,c'},\ c < c'$ be the basis such that $\widetilde{B}_{c,c'} = (\vec{e_i})_{i \in \widetilde{I}_{c,c'}}$, where $\widetilde{I}_{c,c'} = \{i,\ i \in \{1, 2, 3, \dots, 4^k\} \mid T(g_c).\vec{e_i} \neq 0$ and $T(g_{c'}).\vec{e_i} \neq 0$ and $\forall d \neq c,\ d \neq c', T(g_d).\vec{e_i} = 0\}$. $\widetilde{B}_{c,c'}$ contains all dimensions present only in both $T(g_c)$ and $T(g_{c'})$. In other words, $\widetilde{B}_{c,c'}$ contains k-mers specific to the overlap between g_c and $g_{c'}$. Since we assume that the overlap between g_c and $g_{c'}$ defines the centromere, $\widetilde{E}_k^{c,c'} = span(\widetilde{B}_{c,c'})$ is the centromere-specific k-mer space. For convenience in notations, we denote $\widetilde{B}_{c,c'}$ by $\widetilde{B}_d$, where d is an integer such that $n < d \leq n + m$, d is unique to the pair (c, c'), and m is the total number of non-null centromere-specific k-mer spaces ($m \leq n/2$). At most $n/2$ centromeres can be defined given a set of n chromosomal sequences, because some centromeres are not defined in the case of no overlap. In the case there is no overlap, then $m = 0$.

Orthogonal decomposition

The vector spaces $\widetilde{E}_k^c$ allow a decomposition of the k-mer vector space E_k. This section explains the construction of this decomposition. First, we prove the fact that a k-mer from an object s cannot belong to more than one target specific k-mer space.

Claim 1. *For all $(c, c') \in \{1, \dots, n + m\}^2$, $c \neq c'$, we have $\widetilde{E}_k^c \perp \widetilde{E}_k^{c'}$.*

Proof. By construction, for all $\vec{u} \in \widetilde{E}_k^c, \forall \vec{u'} \in \widetilde{E}_k^{c'}$, we have $\vec{u} = \sum_{i \in \widetilde{I}_c} u_i \vec{e_i}$ and $\vec{u'} = \sum_{i \in \widetilde{I}_{c'}} u'_i \vec{e_i}$. Then, $\vec{u}.\vec{u'} = \sum_{i \in \{1,2,3,\dots,4^k\}} u_i u'_i = \sum_{i \in \widetilde{I}_c \cap \widetilde{I}_{c'}} u_i u'_i$. By definition of the basis, $\widetilde{I}_c \cap \widetilde{I}_{c'} = \emptyset$ because $c \neq c'$, so $\vec{u}.\vec{u'} = 0$. □

Since we have established that spaces $\widetilde{E}_k^c$ are pairwise orthogonal, we can define $\widetilde{E}_k$ as the vector space resulting from the direct sum of all $\widetilde{E}_k^c$, i.e.,

$$\widetilde{E}_k = \bigoplus_{c=1}^{n+m} \widetilde{E}_k^c \qquad (3)$$

Since $\widetilde{E}_k$ contains non-null spaces, $\widetilde{E}_k$ is not a null space. Also, since E_k is an Euclidean space and $\widetilde{E}_k \subset E_k$, we can define the orthogonal decomposition of E_k as

$$E_k = \widetilde{E}_k \oplus \widetilde{E}_k^{\perp} \tag{4}$$

where the vector space $\widetilde{E}_k^{\perp}$ represents the space of common k-mers within all targets.

The last two relations are useful when we consider the assignment of an object s to a target sequence g_c. Since $T(s) \in E_k$, Equation 4 suggests that there must exist two unique vectors $\vec{u}$ and $\vec{u}^{\perp}$ such that $T(s) = \vec{u} + \vec{u}^{\perp}$, where $\vec{u}$ is the orthogonal projection of $T(s)$ to $\widetilde{E}_k$, and $\vec{u}^{\perp}$ is the orthogonal projection of $T(s)$ to $\widetilde{E}_k^{\perp}$. In order words, $\vec{u} = T(s)_{/\widetilde{E}_k}$, and $\vec{u}^{\perp} = T(s)_{/\widetilde{E}_k^{\perp}}$. Let us now focus on $\vec{u}$. Equation 3 allows us to decompose this vector by projecting it into each $\widetilde{E}_k^c$:

$$\vec{u} = T(s)_{/\widetilde{E}_k} = \sum_{c=1}^{n+m} T(s)_{/\widetilde{E}_k^c}$$

It follows that

$$\|\vec{u}\|_1 = \left\|T(s)_{/\widetilde{E}_k}\right\|_1 = \sum_{c=1}^{n+m} \left\|T(s)_{/\widetilde{E}_k^c}\right\|_1 \tag{5}$$

where $\left\|T(s)_{/\widetilde{E}_k^c}\right\|_1$ is the count of g_c-specific k-mers in s. As a consequence, projecting the spectrum of any object s to each target-specific space $\widetilde{E}_k^c$ reveals the uniquely shared substring between object s and target c.

Orthogonal projections

Let us now introduce more properties based on the decomposition described above.

Claim 2. *If an object s is not a substring of a target g_c, then* $\left\|T(s)_{/\widetilde{E}_k^c}\right\|_1 = 0$.

Proof. If s is not a substring of $g_c \in \{g_1, g_2, \ldots, g_n\}$ then any k-mer from s cannot be g_c-specific. Therefore, the count g_c-specific k-mers contained in s is 0. The conclusion follows. □

Claim 3. *If s is a repeat of $\{g_1, g_2, \ldots, g_n\}$ and $m = 0$ then, for all $c \in \{1, 2, \ldots, n\}$, we have* $\left\|T(s)_{/\widetilde{E}_k^c}\right\|_1 = 0$.

Proof. Recall that $T(s) = \vec{u} + \vec{u}^{\perp}$ and $\|\vec{u}\|_1 = \sum_{c\in\{1,2,\ldots,n\}} \left\|T(s)_{/\widetilde{E}_k^c}\right\|_1$. For any c, $\left\|T(s)_{/\widetilde{E}_k^c}\right\|_1$ is the count of k-mers specific to g_c contained in s. Since $m = 0$, there is no centromere-specific space. Now, let us assume for some c, $\left\|T(s)_{/\widetilde{E}_k^c}\right\|_1 \neq 0$, this implies (since there is no centromere-specific spaces) that s contains at least one k-mer that is specific to g_c and no other target. So s contains a substring that appears only in one target sequence. In other words, s is not repeated in its entirety, so this contradicts the hypothesis that s is a repeat. This implies that for all $c \in \{1, 2, \ldots, n\}$, we have $\left\|T(s)_{/\widetilde{E}_k}\right\|_1 = 0$. □

Theorem 1. *Given a set of targets $\{g_1, g_2, \ldots, g_n\}$, and a set of objects $\{s_1, s_2, \ldots, s_p\}$, if s_l originates from at least one target in $\{g_1, g_2, \ldots, g_n\}$ and $m = 0$, then there exists at most one index $c^*(1 \leq c^* \leq n)$ such that for all $c \in \{1, 2, \ldots, n\}, c \neq c^*$, $\left\|T(s_l)_{/\widetilde{E}_k^c}\right\|_1 = 0$, where for each target c, $1 \leq c \leq n$, $\widetilde{E}_k^c$ is the g_c-specific k-mer space.*

Proof. Let s_l be a sequence in $\{s_1, s_2, \ldots, s_p\}$. If s_l is a repeat of $\{g_1, g_2, \ldots, g_n\}$ then Claim 3 holds. Then, the conclusion follows. Otherwise, if s_l is not a repeat then s_l is a substring of exactly one sequence g_{c^*}. In addition, $m = 0$ so s_l is not a substring of any sequence other than g_{c^*}. So by Claim 2, for all $c \in \{1, 2, \ldots, n\}, c \neq c^*$, $\left\|T(s_l)_{/\widetilde{E}_k^c}\right\|_1 = 0$. □

When s_l is a substring of exactly one target sequence g_{c^*}, if s_l does not contain any g_{c^*}-specific k-mers then $\left\|T(s_l)_{/\widetilde{E}_k^{c^*}}\right\|_1 = 0$. This may happen when the sequence s is too short to capture any g_{c^*}-specific k-mers or if k is too small.

However, if $\left\|T(s_l)_{/\widetilde{E}_k^{c^*}}\right\|_1 \neq 0$ then the origin of the sequence s is g_{c^*}.

Theorem 2. *Given a set of targets $\{g_1, g_2, \ldots, g_n\}$, and a set of objects $\{s_1, s_2, \ldots, s_p\}$, if sequence s_l originates from at least one target in $\{g_1, g_2, \ldots, g_n\}$ and targets are chromosome arms such that $m \neq 0$, then there exists at most two distinct indexes $(d, e) \in \{1, 2, \ldots, n+m\}^2$ such that for all $c \in \{1, 2, \ldots, n+m\}, c \neq d, c \neq e$, $\left\|T(s_l)_{/\widetilde{E}_k^c}\right\|_1 = 0$.*

Proof. We consider four cases. In the first case s_l is a repeat and is a substring of at least three targets then s_l can not contain any specific k-mer to any target. Then, for all $c \in \{1, 2, \ldots, n+m\}$, we have $\left\|T(s)_{/\widetilde{E}_k^c}\right\|_1 = 0$. The conclusion follows.

In the second case, s_l is a repeat and is a substring of two targets g_{c_1} and g_{c_2}. Assume that g_{c_1} and g_{c_2} are the arms of the same chromosome. If s_l is a substring in the overlap between g_{c_1} and g_{c_2} then s_l can contain k-mers specific to the corresponding centromere. It follows that there exists $d \in \{n+1, \ldots, n+m\}$ such that $\left\|T(s_l)_{/\widetilde{E}_k^d}\right\|_1$ may be not null, however for all $c \in \{1, 2, \ldots, n+m\}, c \neq d$, we have $\left\|T(s)_{/\widetilde{E}_k^c}\right\|_1 = 0$ and the conclusion follows. Otherwise, s_l is a repeat and any k-mer in s_l can not be specific to any target (because it appears twice) or any centromere (because it is not in the overlap). So for all $c \in \{1, 2, \ldots, n+m\}$, we have $\left\|T(s)_{/\widetilde{E}_k^c}\right\|_1 = 0$. The conclusion follows. If g_{c_1} and g_{c_2} are not the arms of the same chromosome then s_l is merely a repeat, therefore the conclusion follows.

In the third case s_l is a not repeat (s_l originates from g_d) and is a partial substring of the overlap between two targets g_d and $g_{d'}$. Say g_d and $g_{d'}$ are the arms of the same chromosome. Then, s_l can contain g_d-specific k-mers, and also k-mers specific to the centromere (formed by g_d and $g_{d'}$), and no other k-mers specific to other space. It follows that $\left\|T(s_l)_{/\widetilde{E}_k^d}\right\|_1$ may be not null and there exists $e \in \{n+1, \ldots, n+m\}$ such that $\left\|T(s_l)_{/\widetilde{E}_k^e}\right\|_1$ may be not null either. Thus, the conclusion follows. If g_d and $g_{d'}$ are not the arms of the same chromosome, then s_l can only contain g_d-specific k-mers, and the conclusion follows.

In the fourth case, s_l is not a repeat and is not a substring (nor a partial substring) of any overlap between two targets then s_l is a substring of exactly one sequence g_{c^*}. Similarly as the previous proof, the conclusion follows. □

Theorems 1 and 2 show that, given an object s the projections of $T(s)$ on all targets-specific spaces are guaranteed to be null, except for the one that is related to the origin of s. As a consequence, if a sequence s is known to be a substring of at most one target in $\{g_1, g_2, \ldots, g_n\}$, then the problem of assigning s is reduced to the problem of studying non-null projections of $T(s)$ on the $n+m$ specific vector spaces.

Assignment method

The two previous theorems lay the theoretical foundation on which CLARK's assignment method was designed. Given an object s, CLARK first computes the projections of the spectrum $T(s)$ on all targets-specific spaces.

If the number of non-null projection is zero, then object s is not assigned (a higher value of k might be necessary). If there is exactly one non-null projection, say $\left\|T(s)_{/\widetilde{E}_k^c}\right\|_1 \neq 0$, for some c, then object s contains g_c-specific k-mers. In this case, CLARK assigns object s to target c. If there is more that one non-null projections, say $\left\|T(s)_{/\widetilde{E}_k^c}\right\|_1 \neq 0$, for $c = c_1$ and $c = c_2$, we expect c_1 and c_2 to be arms of the same chromosome. In this case, the chromosome is identified, and s is assigned to c_1 if $\left\|T(s)_{/\widetilde{E}_k^{c_1}}\right\|_1 > \left\|T(s)_{/\widetilde{E}_k^{c_2}}\right\|_1$, otherwise s is assigned to c_2.

The previous cases can be summarized by the following rule. First compute

$$c^* = \arg \max_{1 \leq c \leq n+m} \left\|T(s)_{/\widetilde{E}_k^c}\right\|_1 \tag{6}$$

then object s is assigned to target c^*.

Theoretically, one should expect a large number of projections to be zero. In practice, with real (noisy) data null-expected projections will have instead low counts. In other words, instead of expecting up to two non-null projections, we should expect up to two projections having high 1-norm compared to all others.

Given a object s, CLARK computes the highest norm, namely $\left\|T(s)_{/\widetilde{E}_k^{c*}}\right\|_1$, and the second highest norm, namely $\left\|T(s)_{/\widetilde{E}_k^{c**}}\right\|_1$. Then, CLARK evaluates the confidence of the assignment by using the following confidence score, which ranges from 0.5 to 1.

$$\text{confidence} = \frac{\left\|T(s)_{/\widetilde{E}_k^{c*}}\right\|_1}{\left\|T(s)_{/\widetilde{E}_k^{c*}}\right\|_1 + \left\|T(s)_{/\widetilde{E}_k^{c**}}\right\|_1}$$

Another useful statistic is $\gamma = \sum_{1 \leq c \leq n+m} \left\|T(s)_{/\widetilde{E}_k^c}\right\|_1 / \left\|T(s)\right\|_1$, which indicates the proportion of k-mers hitting all targets.

CLARK's algorithm

Given a set of targets $\{g_1, g_2, \ldots, g_n\}$, a set of objects $\{s_1, s_2, \ldots, s_p\}$ and an integer k, CLARK's computes for each object s (1) the top two target assignments, (2) the confidence score, (3) the number of hits against each target and (4) γ.

To achieve efficient computations, we use a hash table to store all k-mers from the targets. This data structure allows one to remove all common k-mers, and also performs fast queries (constant time, on average). We have designed our own hash table of size L based on a chaining structure. The hash function h is defined as follows. Given a k-mer km represented by a number l, where $l = \sum_{i=1}^{k} a[i]4^{i-1}$ (with $a[i] = 0$ if $km[i] = A$, $a[i] = 1$ for C, $a[i] = 2$ for G and $a[i] = 3$ for T or U), we define $h(l) = l \mod L$, where L is defined below. To reduce the amount of bits to be stored per k-mer, we only save in the hash table the

value l/L for bucket $h(l)$. Indeed, since L is known, $h(l)$ and l/L contain enough information to compute back l because $l = (l/L) \times L + h(l)$. If $k = 31$ and $L > 4^{15}$ then (l/L) can be stored in four bytes. As a consequence, any 31-mer can be represented with only four bytes instead of eight. If $k \leq 23$ and $L > 4^{15}$ then two bytes are enough to store any k-mer; if $k \leq 19$ and $L > 4^{15}$ then only one byte is enough.

The implementation of our algorithm using a hash table is illustrated in Table S5.

Supplementary Note 2: Confidence score analysis

Our software tool CLARK, unlike other most of other sequence classifiers, provides confidence scores. Here we want to study the relation between confidence scores and correctness of results.

Figure S1 shows the distribution of the number of assignments as a function of the confidence score for all the datasets presented in this study, namely barley BACs and unigenes (A2A), barley BACs (R2R and A2A), and the four metagenomic datasets ("HiSeq", "MiSeq", "simBA-5", and "simHC.20.500"). Observe the high density of high confidence assignments in all cases, especially for "HiSeq" and "MiSeq" datasets. For all these datasets, when running CLARK in full mode, we observe that at least 95% of all assignments have confidence score higher or equal than 0.98. This is clear evidence that, in the full mode, conflicts in the classification rarely occur.

Figure S2 shows the proportion of correct assignments (y-axis) as a function of confidence score ranges (x-axis). Observe that at least 95% of assignments having confidence of 0.90 or higher are correct.

References

[1] COMPEAU, P. E., PEVZNER, P. A., AND TESLER, G. How to apply de Bruijn graphs to genome assembly. *Nature biotechnology 29*, 11 (2011), 987–991.

	Installation/Database construction			Classification
	Time (HH:MM)	RAM Peak usage (GB)	Memory Disk (GB)	RAM Peak usage (GB)
NBC	19:10	< 1	52.0	< 1
KRAKEN	06:07	167.9	141.0	77.7
CLARK	02:45	164.1	42.4	70.1
CLARK-l	00:05	3.8	< 1	2.8

Table S1: Details of the time and memory usage (RAM and disk) for the installation (or database construction of the 2,752 bacterial genomes of NCBI/RefSeq), and the classification of NBC, KRAKEN and CLARK, at the genus-level and in default mode. Measurements of the installation time and RAM peak usage are done for NBC, KRAKEN and CLARK using default settings and single-thread. RAM peak usage was obtained by the attribute `maximum resident set size` of the command `/usr/bin/time -v` available on Linux platforms.

	"HiSeq" dataset			
Number of threads	1	2	4	8
KRAKEN	2,332	3,647	3,534	3,876
CLARK	3,116	5,484	9,626	15,807
KRAKEN-Q	6,224	7,712	7,693	7,506
CLARK-E	32,450	39,841	46,386	52,896
	"MiSeq" dataset			
Number of threads	1	2	4	8
KRAKEN	1,361	2,038	3,605	3,616
CLARK	1,670	3,040	4,905	8,120
KRAKEN-Q	5,308	5,553	8,362	8,642
CLARK-E	28,988	32,199	41,970	49,383

Table S2: Genus-level classification speed (expressed as 10^3 reads/min) as a function of the number of threads ($k = 31$).

Targets	*19-mers*	*discriminative 19-mers*	*assignments*	*low confidence*	*high confidence*
1H	180,176,713	108,894,740	2,111	7.1%	92.9%
2HC	-	814,357	0	-	-
2HL	103,679,920	64,700,161	1,424	3.4%	96.6%
2HS	90,912,314	54,449,430	1,071	3.5%	96.5%
3HC	-	1,532,968	0	-	-
3HL	123,140,951	78,158,244	1,411	3.3%	96.7%
3HS	111,951,787	70,473,478	897	5.5%	94.5%
4HC	-	3,105,047	56	67.9%	32.1%
4HL	106,999,773	64,749,958	1,132	3.5%	96.5%
4HS	89,027,872	51,612,790	890	4.4%	95.6%
5HC	-	604,030	0	-	-
5HL	117,915,094	77,128,375	1,658	2.8%	97.2%
5HS	58,067,400	34,037,607	654	5.4%	94.6%
6HC	-	469,530	0	-	-
6HL	74,485,223	44,221,184	1,132	3.4%	96.6%
6HS	111,834,123	83,957,421	846	6.5%	93.5%
7HC	-	795,923	0	-	-
7HL	92,603,503	58,159,248	1,179	3.6%	96.4%
7HS	90,217,777	55,276,671	1,234	4.8%	95.2%
Total	1,351,012,450	853,141,162	15,695	4.6%	95.4%

Table S3: Summary of CLARK's assignment of 15,695 BACs (represented as assemblies) to barley chromosome arms (assemblies) and centromeres ($k = 19$). Columns: (1) barley chromosome 1H, twelve chromosome arms, and six centromeres; (2) number of distinct k-mers in each target; (3) number of discriminative k-mers present in target sequences (must occur at least once); (4) number of assigned objects per target; (5) number of low confidence assignment per target; (6) number of high confidence assignment per target; (7) percentage of low confidence assignment (as a fraction of the total number of assigned objects per target); (8) percentage of high confidence assignment (as a fraction of the total number of assigned objects per target).

IMG Taxon ID	Genome
640753002	*Alkaliphilus metalliredigens* QYMF
640427103	*Bradyrhizobium sp.* BTAi1
637000047	*Burkholderia cepacia* AMMD
637000160	*Chelativorans sp.* BNC1
640069309	*Clostridium thermocellum* ATCC 27405
637000088	*Dechloromonas aromatica* RCB
643348537	*Desulfitobacterium hafniense* DCB-2
637000116	*Frankia sp.* CcI3
637000119	*Geobacter metallireducens* GS-15
639633037	*Marinobacter aquaeolei* VT8
637000162	*Methanosarcina barkeri Fusaro*, DSM 804
637000192	*Nitrobacter hamburgensis* X14
639633046	*Nocardioides sp.* JS614
637000208	*Polaromonas sp.* JS666
637000216	*Pseudoalteromonas atlantica* T6c
637000221	*Pseudomonas fluorescens* Pf0-1
640069327	*Rhodobacter sphaeroides* 2.4.1, ATCC BAA-808
637000260	*Shewanella sp.* MR 7
639633063	*Syntrophobacter fumaroxidans* MPOB

Table S4: Genomes used in the “simHC.20.500” dataset (JGI database).

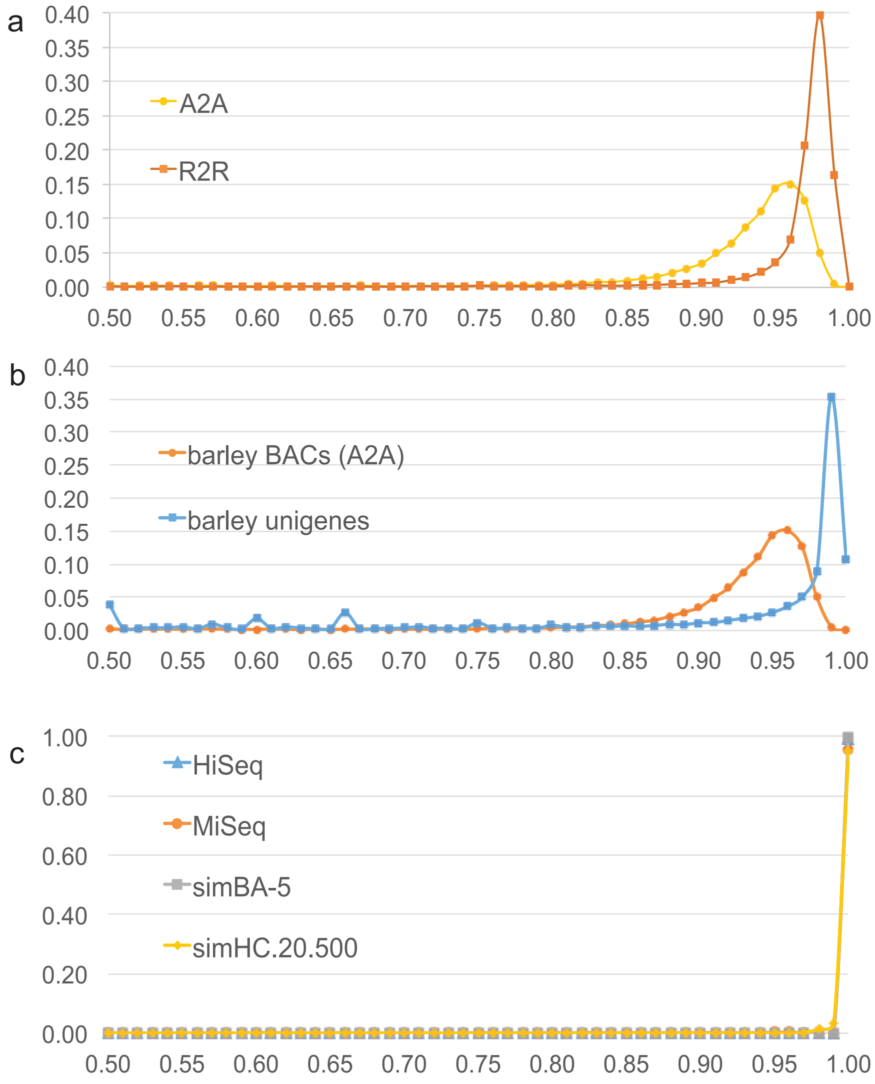

Figure S1: Distribution of the number of assignments as a function of the confidence score for (a) barley BACs (R2R) and (A2A) (b) barley unigenes and BACs (A2A) and (c) the four simulated metagenome sets ("HiSeq", "MiSeq", "simBA-5", and "simHC.20.500").

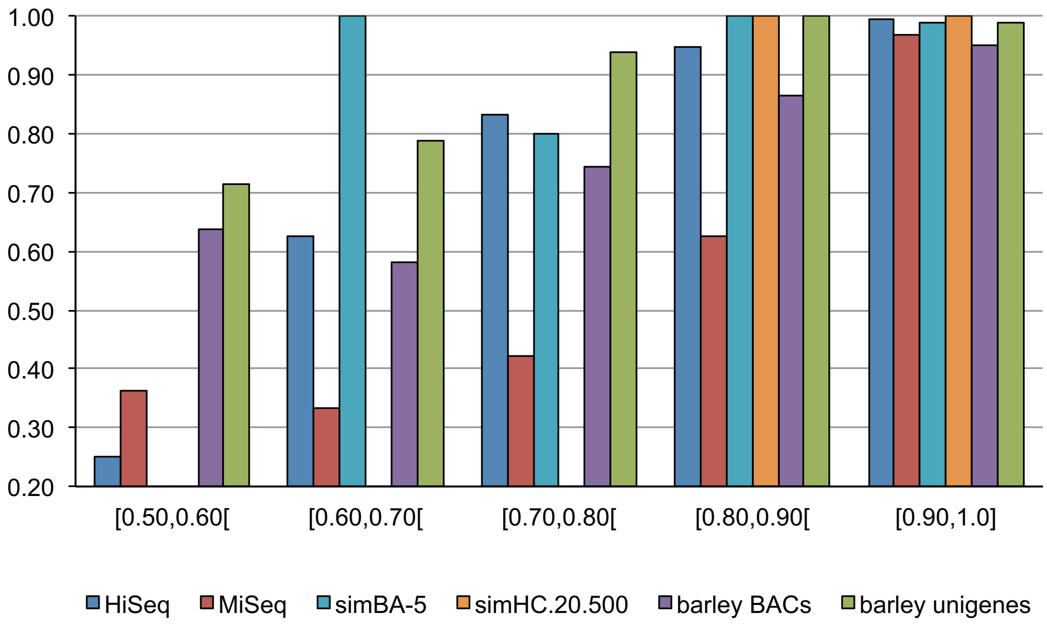

Figure S2: Probability (y-axis) of a correct assignment for a particular range of CLARK's confidence scores (x-axis).

	Input: integer k, n target sequences $(g_c)_{1 \leq c \leq n}$, p object sequences $(s_l)_{1 \leq l \leq p}$
1	if hash table H related to $(T(g_c))_{1 \leq c \leq n}$ already exists then
2	load H
3	goto 15
4	create an empty hash table H
5	for all $c, 1 \leq c \leq n$:
6	for each $(km, w) \in T(g_c)$:
7	if ($km \in H$) then
8	update the list of targets associated to km by adding c and increase the occurrence of km by w
	else
9	insert (km, w, c) in H
10	for each $km \in H$:
11	if the list of targets for km has more than three elements then
12	remove km from H
	else
13	if the list of origins for km has exactly two elements $(c_1, c_2, c_1 < c_2)$ and from different chromosomes then
14	remove km from H
15	for all $l, 1 \leq l \leq p$:
16	if $T(s_l) = 0$ then
17	output l, “not assigned”
	continue
18	create $n + m$ empty bins: $b_1, b_2, \ldots, b_n, \ldots, b_{n+m}$
19	for each $(km, w) \in T(s_l)$:
20	if $km \in H$ (in target c) then
21	$b_c = b_c + w$
22	c^* = $\arg\max\{b_1, b_2, \ldots, b_n, \ldots, b_{n+m}\}$
23	c^{**} = $\arg\max\{\{b_1, b_2, \ldots, b_n, \ldots, b_{n+m}\} - \{b_{c^*}\}\}$
24	$\gamma = \sum_{1 \leq t \leq n+m} b_t / T(s_l)$
25	If $\gamma = 0$ then
26	Output l, “not assigned”
	continue
27	$confidence = \frac{b_{c^*}}{b_{c^*} + b_{c^{**}}}$
28	output l, $b_1, b_2, \ldots, b_{n+m}, \gamma,\ c^*$, c^{**}, $confidence$

Table S5: Description of CLARK’s algorithm (“full” mode)
